# Supplementary material for: Combined and differential roles of ADD domains of DNMT3A and DNMT3L on DNA methylation landscapes in mouse germ cells
Source: Nat Commun. 2024 Apr 16;15:3266. doi: 10.1038/s41467-024-47699-2 (PMC11021467; doi:10.1038/s41467-024-47699-2)
Supplement: Supplementary file 1 — Supplementary Information [file 41467_2024_47699_MOESM1_ESM.pdf]

Supplementary Information

Combined and differential roles of ADD domains of DNMT3A and DNMT3L on DNA methylation landscapes in mouse germ cells

Naoki Kubo[1]*, Ryuji Uehara[1], Shuhei Uemura[1,3], Hiroaki Ohishi[2], Kenjiro Shirane[3] and Hiroyuki Sasaki[1]*

[1]Division of Epigenomics and Development, Medical Institute of Bioregulation, Kyushu University, Fukuoka 812-8582, Japan

[2]Division of Gene Expression Dynamics, Medical Institute of Bioregulation, Kyushu University, Fukuoka 812-8582, Japan

[3]Department of Genome Biology, Graduate School of Medicine, Osaka University, Osaka 565-0871, Japan

*Corresponding author and lead contact

E-mail: naoki.kubo@bioreg.kyushu-u.ac.jp (N. Kubo) and hsasaki@bioreg.kyushu-u.ac.jp (H. Sasaki)

Supplementary Figure 1

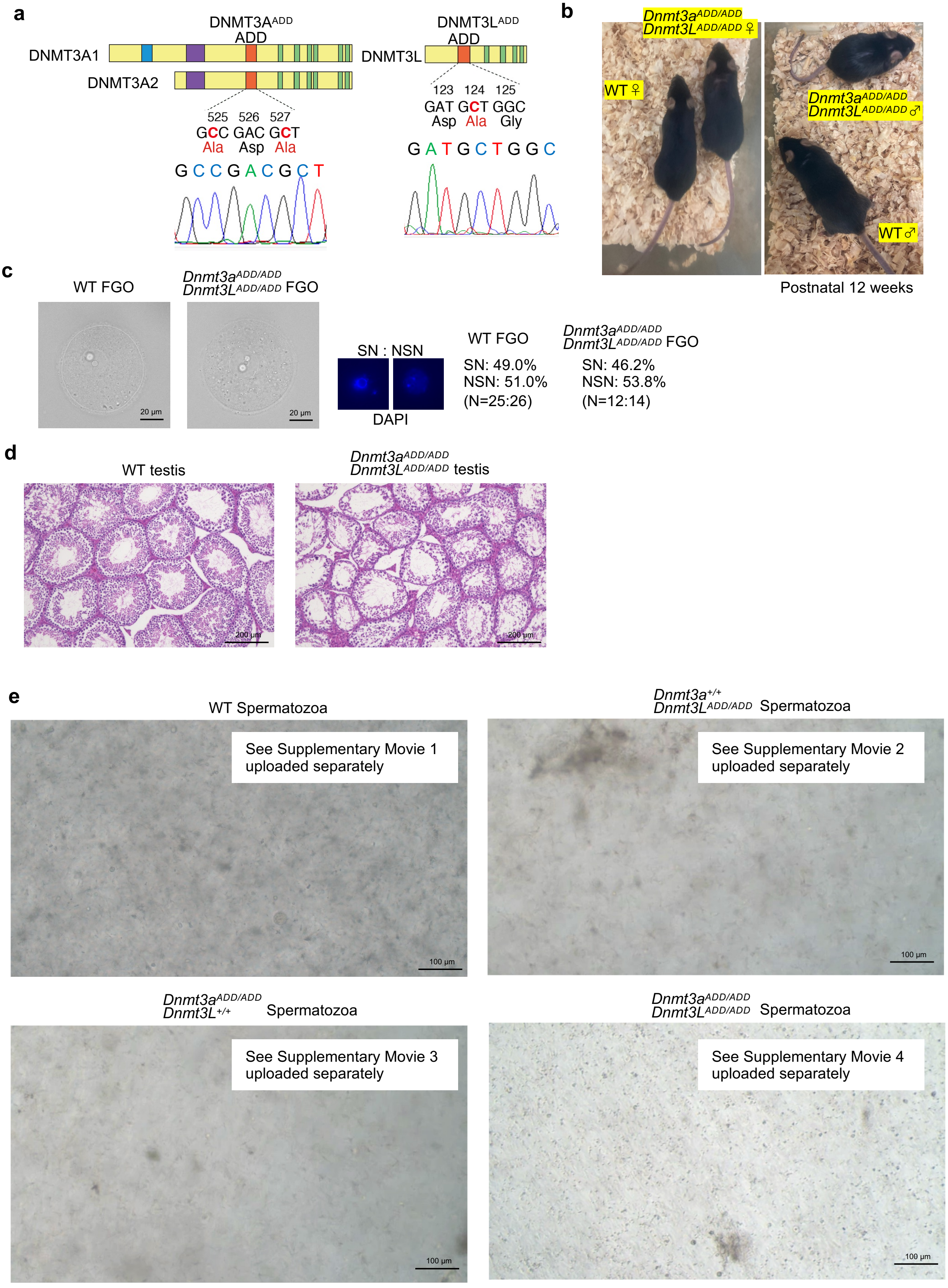

Supplementary Figure 1 | Phenotypes of mice and gametes carrying ADD mutations in DNMT3A and DNMT3L.

a, Genotyping by Sanger sequencing of DNMT3A^{ADD} and DNMT3L^{ADD}.

b, Representative images of 12 weeks old adult mice of wild-type and [*Dnmt3a*$^{ADD/ADD}$, *Dnmt3L*$^{ADD/ADD}$].

c, Representative images of wild-type and [*Dnmt3a*$^{ADD/ADD}$, *Dnmt3L*$^{ADD/ADD}$] FGO, and the proportions of SN-type versus NSN-type FGOs (putative nuclear maturation rate, SN is the more mature one). Representative DAPI-stained SN-type (surrounded nucleolus) and NSN-type (non-surrounded nucleolus) images are also shown.

d, Hematoxylin-eosin stained sections of wild-type and [*Dnmt3a*$^{ADD/ADD}$, *Dnmt3L*$^{ADD/ADD}$] testis.

e, Movie shots of spermatozoa derived from epididymis of the indicated genotypes (see the movie files uploaded separately; Supplementary Movie 1-4).

Supplementary Figure 2

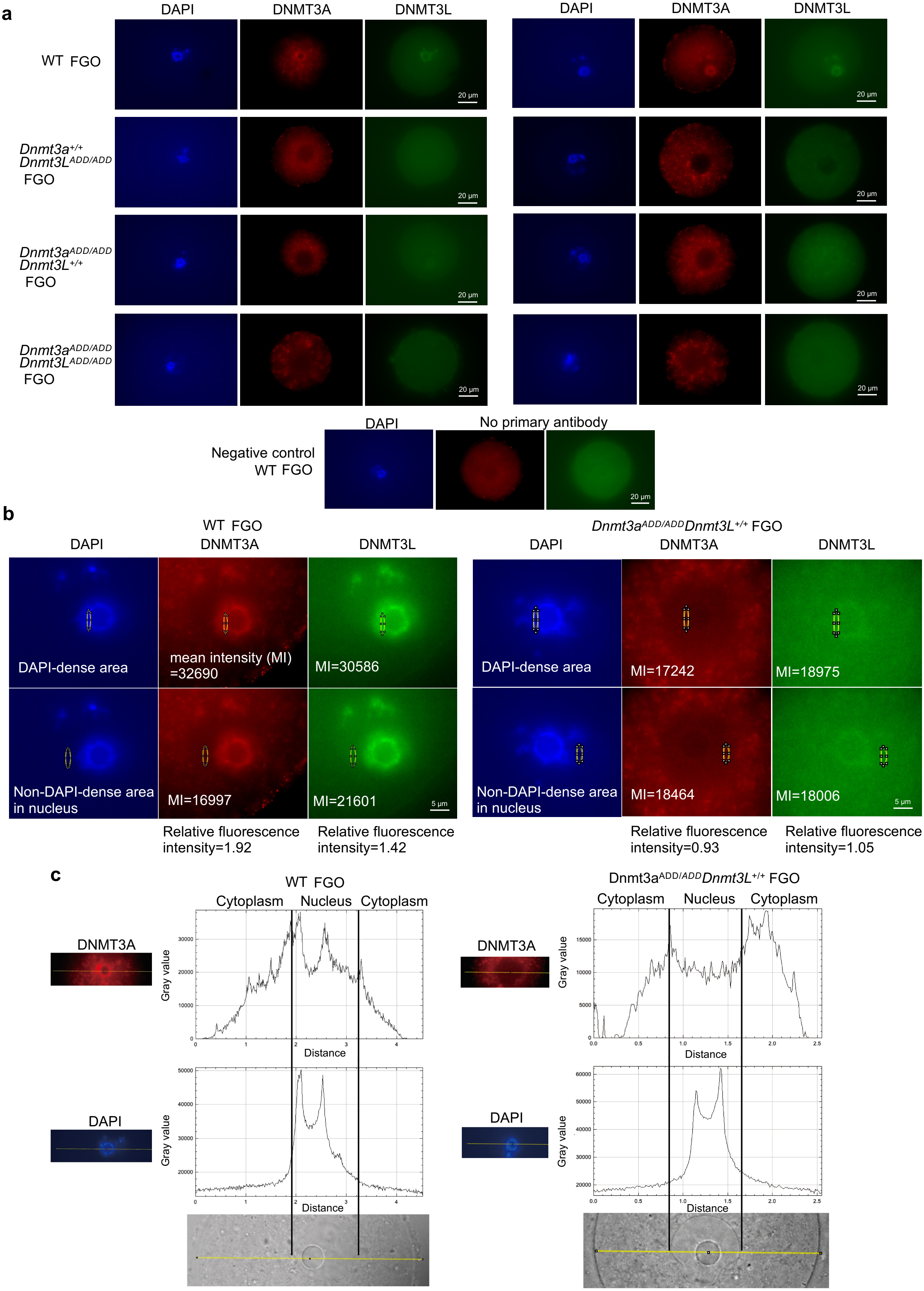

Supplementary Figure 2 | Immunofluorescence staining of FGOs for DNMT3A and DNMT3L.

a, Replicate assays of immunofluorescence staining of wild-type, *Dnmt3L*$^{ADD/ADD}$, *Dnmt3a*$^{ADD/ADD}$, and [*Dnmt3a*$^{ADD/ADD}$, *Dnmt3L*$^{ADD/ADD}$] FGO for DNMT3A (red) and DNMT3L (green). A negative control stained without primary antibody is also shown at the bottom.

b, Examples for the calculation of the relative fluorescence intensity of DNMT3A or DNMT3L in FGO nucleus. Mean intensities on chromatin (DAPI dense region) and nearby non-DAPI regions in the same nuclei were calculated using ImageJ.

c, Fluorescence intensity as a gray value of DNMT3A (top) and DAPI (bottom) across the line (yellow) on cytoplasm and nucleus in WT FGO (left) and *Dnmt3a*$^{ADD/ADD}$ FGO (right). Images of the bright field at the same scale are on the bottom.

Supplementary Figure 3

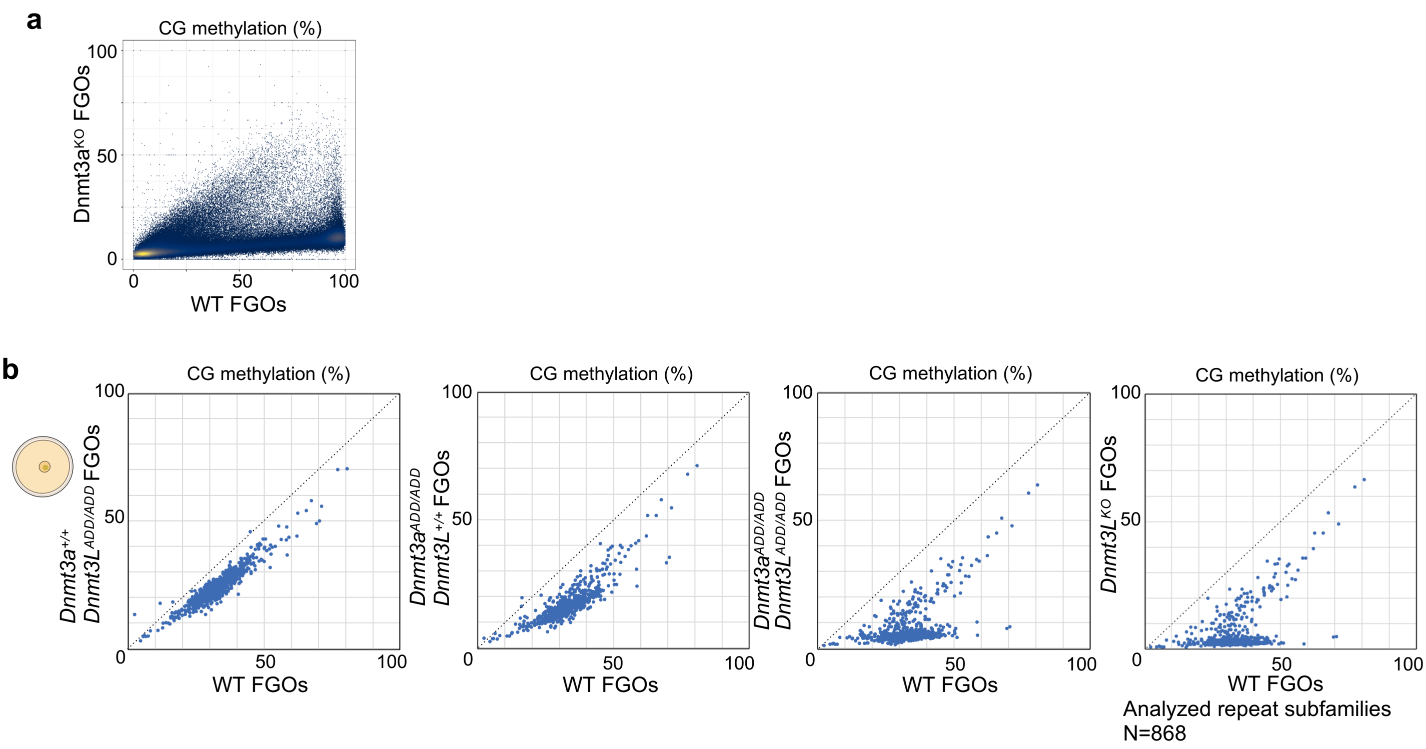

Supplementary Figure 3 | CG methylation on repeat elements in *Dnmt3a*$^{ADD/ADD}$ and/or *Dnmt3L*$^{ADD/ADD}$ FGOs.

a, Scatter plots comparing CG methylation levels of 10-kb genomic bins between *Dnmt3a* knockout and wild-type FGOs.

b, Scatter plots showing CG methylation levels of each repeat element subfamily in FGOs of the indicated genotypes. The number of analyzed repeat subfamilies is 868.

Source data are provided as a Source Data file.

Supplementary Figure 4

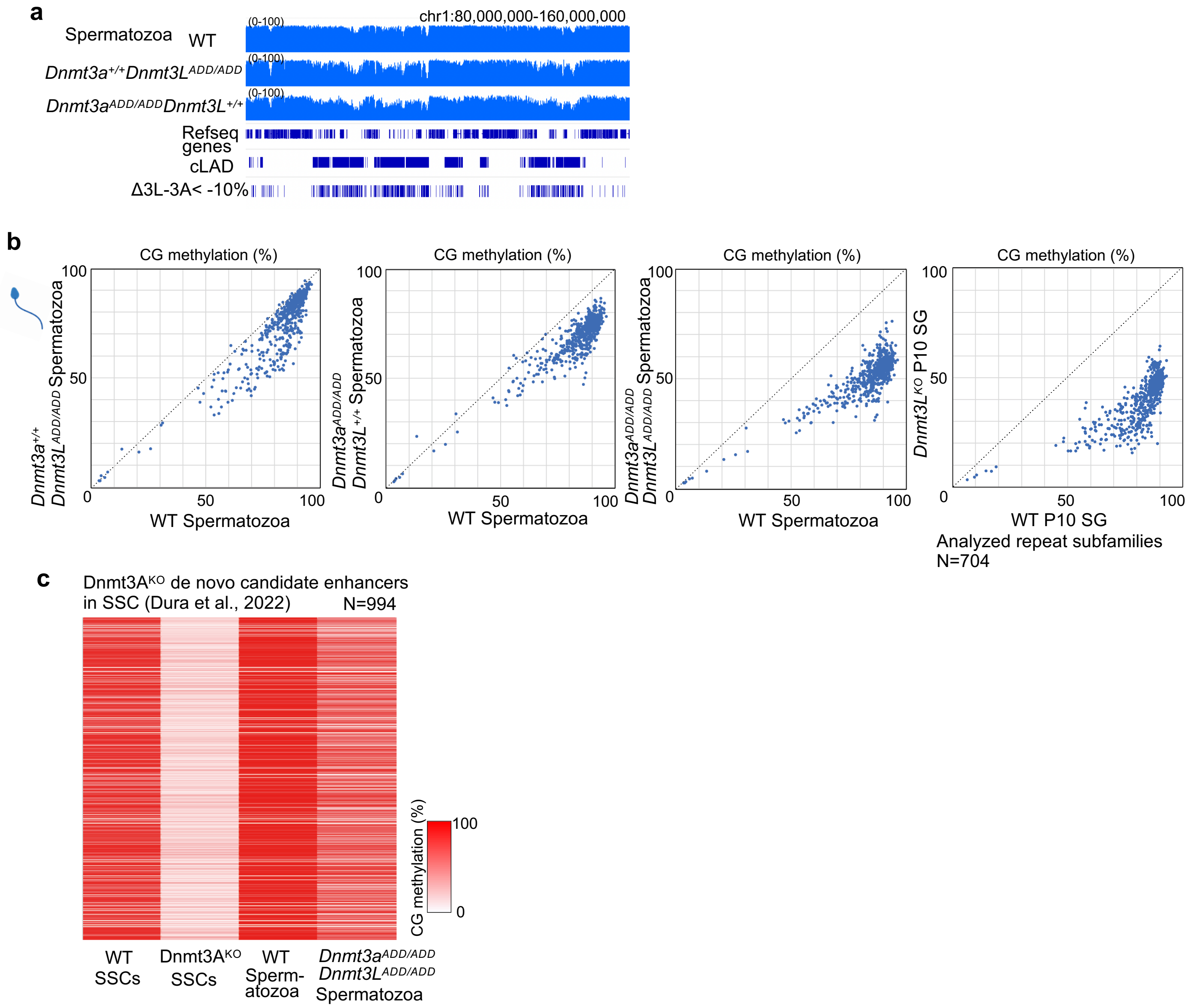

Supplementary Figure 4 | CG methylation on repeat elements and Dnmt3A^{KO} SCC-specific de novo candidate enhancers in *Dnmt3a$^{ADD/ADD}$* and/or *Dnmt3L$^{ADD/ADD}$* spermatozoa.

a, Genome browser view of CG methylation levels of 10-kb bins in spermatozoa (bottom) of the indicated genotypes. Constitutive lamina-associated domains (cLADs) and regions where CG methylation levels in *Dnmt3L$^{ADD/ADD}$* spermatozoa were at least 10% lower than those in Dnmt3a$^{ADD/ADD}$ spermatozoa are shown on the bottom.

b, Scatter plots showing CG methylation levels of each repeat element subfamily in spermatozoa of the indicated genotypes.

c, Heatmap showing CG methylation levels at cis-regulatory elements methylated by DNMT3A that were activated in *Dnmt3A* knockout spermatogonial stem cells (SSCs) [1] in the indicated male germ cells.

Source data are provided as a Source Data file.

Supplementary Figure 5

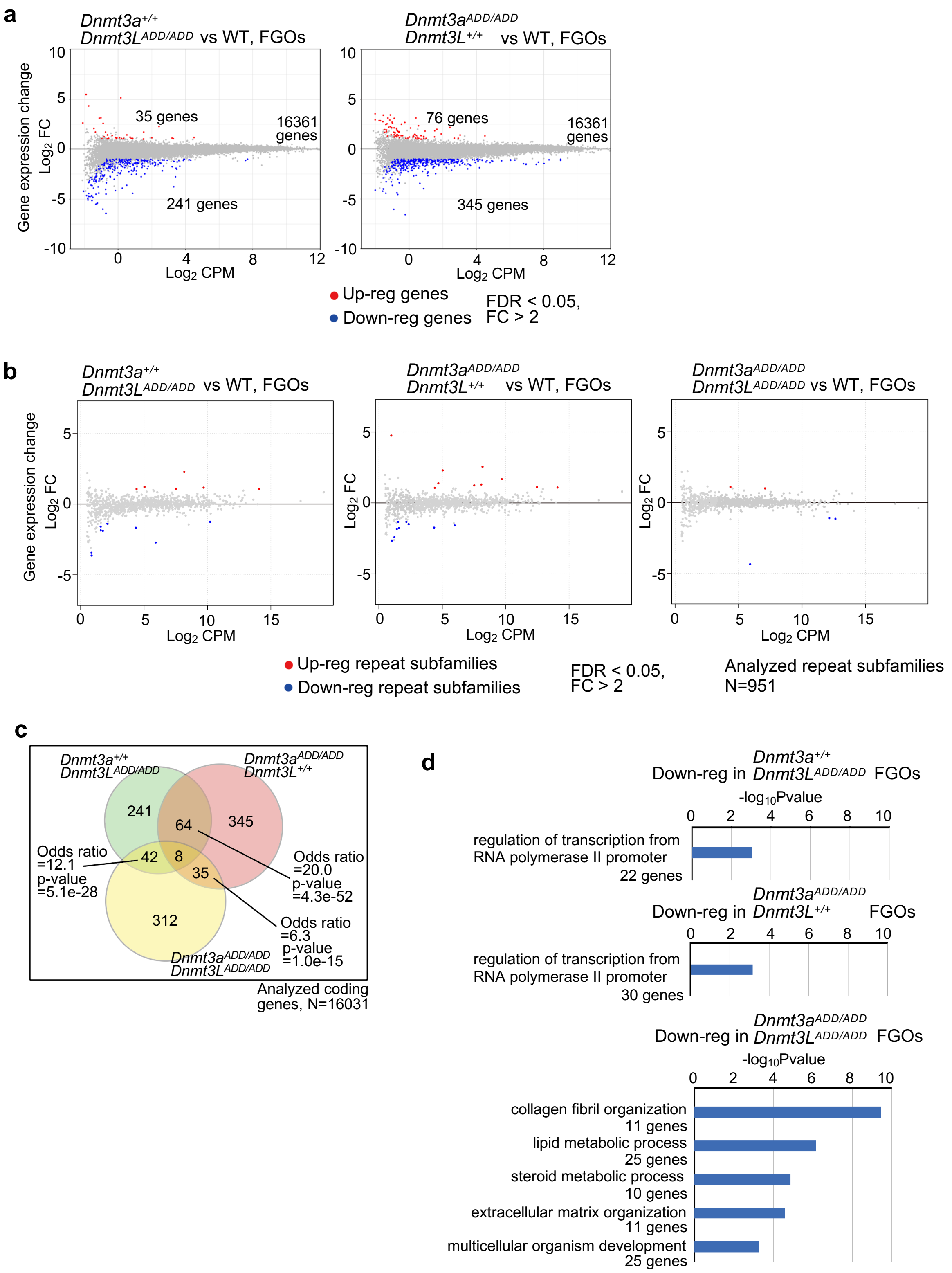

Supplementary Figure 5 | Transcription of genes and repetitive elements in *Dnmt3a*$^{ADD/ADD}$ and/or *Dnmt3L*$^{ADD/ADD}$ germ cells.

a, Gene expression changes between wild-type and *Dnmt3L*$^{ADD/ADD}$ FGOs (left), and changes between wild-type and *Dnmt3a*$^{ADD/ADD}$ FGOs (right). Differentially up-regulated and down-regulated genes in mutated FGOs are plotted in red and blue, respectively (fold-change > 2, FDR < 0.05).

b, Changes of transcription levels of repeat subfamilies between wild-type FGOs and FGOs of the indicated genotypes. Differentially up-regulated and down-regulated repeat elements in mutated FGOs are plotted in red and blue, respectively (fold-change > 2, FDR < 0.05).

c, Venn diagram showing the overlaps among the differentially down-regulated genes of the three types of mutant FGOs. Odds ratios and overlapping p-values are also shown.

d, GO analysis of down-regulated genes in *Dnmt3a*$^{ADD/ADD}$, *Dnmt3L*$^{ADD/ADD}$*, and* [*Dnmt3a*$^{ADD/ADD}$, *Dnmt3L*$^{ADD/ADD}$] FGOs. Enriched GO terms of "Biological Process" with p-values (modified fisher's exact test, DAVID [2]) lower than 1.0×10^{-2} that have more than 10 related genes are shown.

Supplementary Figure 6

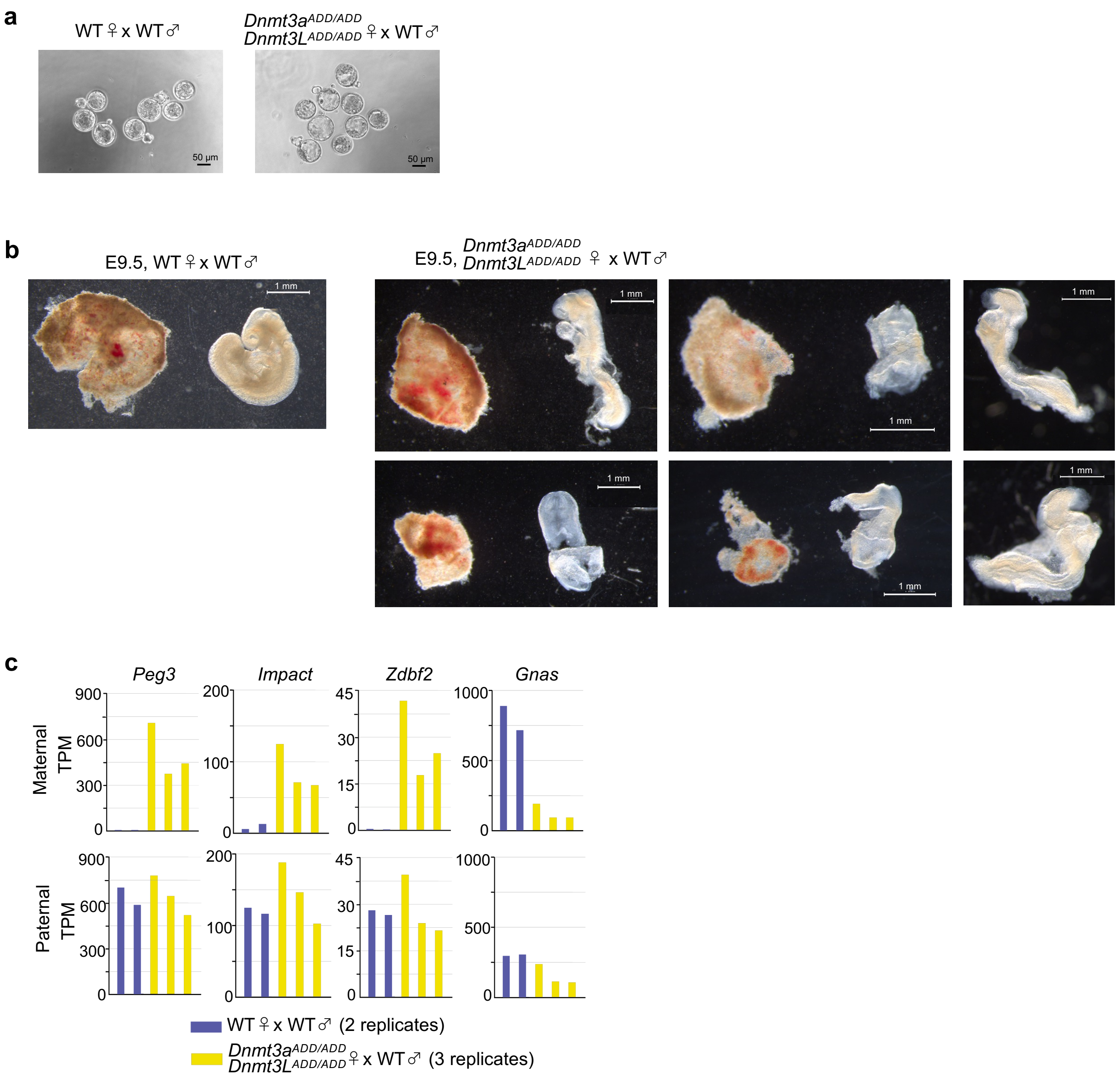

Supplementary Figure 6 | Early embryonic development from the double mutant FGOs.

a, Images of blastocysts obtained from wild-type oocytes and spermatozoa (left) and that from [*Dnmt3a*$^{ADD/ADD}$, *Dnmt3L*$^{ADD/ADD}$] oocytes and wild-type spermatozoa (right) at day 5 after *in vitro* fertilization.

b, Images of embryos and placentas recovered at E9.5. Wild-type embryo with its placenta (left) and embryos and placentas obtained from [*Dnmt3a*$^{ADD/ADD}$, *Dnmt3L*$^{ADD/ADD}$] oocytes and wild-type spermatozoa (right).

c, Allelic expression of representative genes regulated by maternally methylated ICRs in wild-type and [*Dnmt3a*$^{ADD/ADD}$, *Dnmt3L*$^{ADD/ADD}$] embryos. TPM, transcripts per million.

Supplementary Figure 7

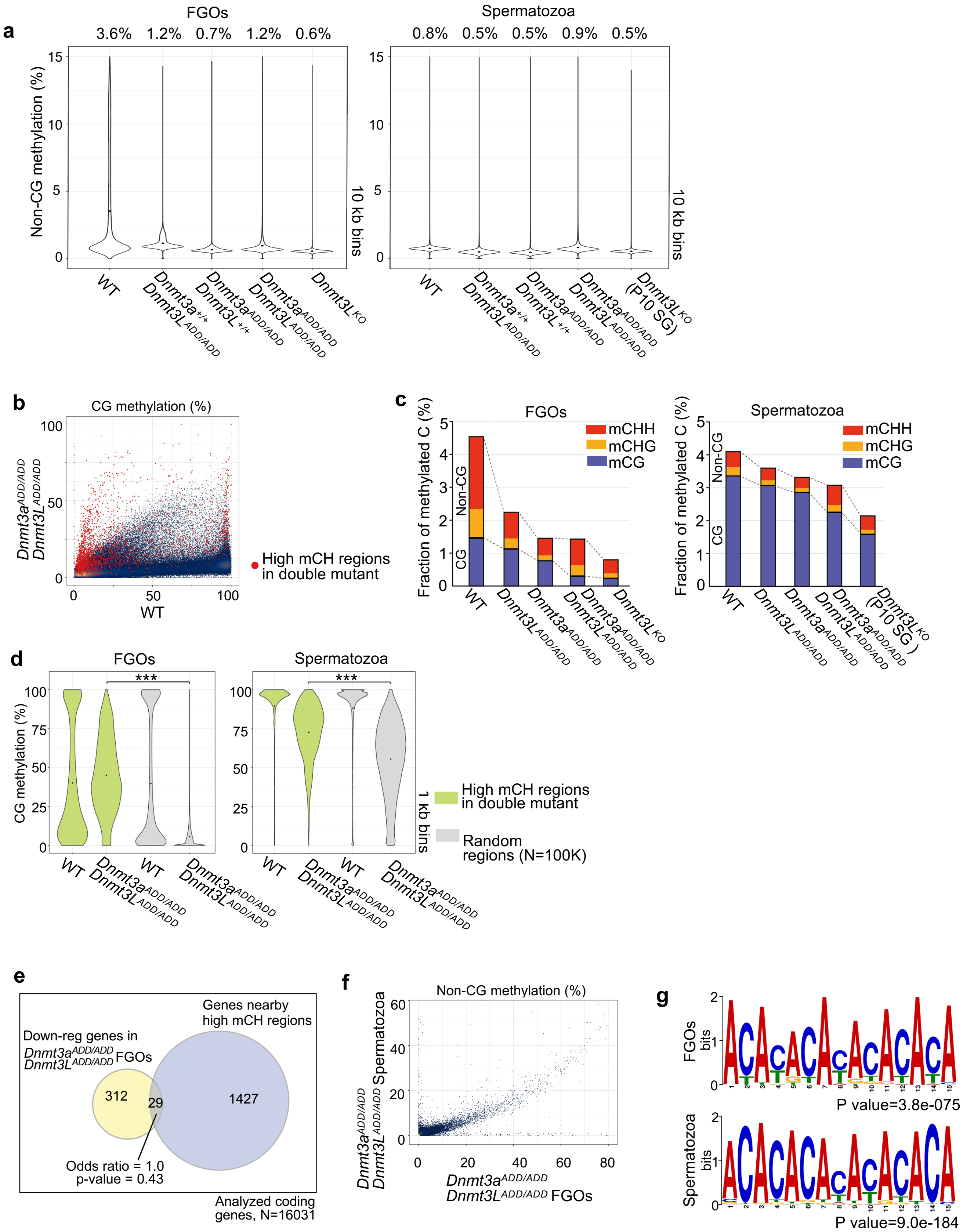

Supplementary Figure 7 | Analysis of non-CG methylation in the double mutant oocytes and spermatozoa.

a, Violin plots showing distributions of non-CG methylation levels of 10-kb bins in FGOs (left) and spermatozoa (right) of the indicated genotypes. The numbers in each plot indicate the global non-CG methylation (mCH) levels.

b, The 10-kb bins showing high non-CG methylation levels in [*Dnmt3a*$^{ADD/ADD}$, *Dnmt3L*$^{ADD/ADD}$] FGOs (> 5% higher than wild-type control) are indicated as red dots and overlaid on the scatter plots shown in Fig. 3a (wild-type versus [*Dnmt3a*$^{ADD/ADD}$, *Dnmt3L*$^{ADD/ADD}$]).

c, Bar graphs showing the fractions of methylated cytosines in all cytosines for FGOs (left) and spermatozoa (right) of the indicated genotypes. Each bar is divided into subsegments representing mCH and mCG, and mCH is further divided into mCHH and mCHG.

d, Violin plots showing distributions of CG methylation levels in 1-kb bins. The 1-kb bins in wild-type and [*Dnmt3a*$^{ADD/ADD}$, *Dnmt3L*$^{ADD/ADD}$] FGOs and spermatozoa were classified into the high-mCH regions (green) and randomly selected regions (gray). *** p value < 0.001 by two-tailed t-test.

e, Venn diagram showing the overlap between the genes down-regulated in [*Dnmt3a*$^{ADD/ADD}$, *Dnmt3L*$^{ADD/ADD}$] FGOs and those nearby the high-mCH regions (distance from the TSS ≤1 kb; 1427 genes). The odds ratio and overlapping p value are also shown.

f, Scatter plots comparing non-CG methylation levels of 10-kb genomic bins between [*Dnmt3a*$^{ADD/ADD}$, *Dnmt3L*$^{ADD/ADD}$] FGOs and [*Dnmt3a*$^{ADD/ADD}$, *Dnmt3L*$^{ADD/ADD}$] spermatozoa.

g, Motifs showing enrichment in high-mCH regions identified separately in [*Dnmt3a*$^{ADD/ADD}$, *Dnmt3L*$^{ADD/ADD}$] FGOs (top) and spermatozoa (bottom).

Source data are provided as a Source Data file.

Supplementary References

1. Dura, M. *et al.* DNMT3A-dependent DNA methylation is required for spermatogonial stem cells to commit to spermatogenesis. *Nat Genet* 54, 469-480 (2022).
2. Sherman, B.T. *et al.* DAVID: a web server for functional enrichment analysis and functional annotation of gene lists (2021 update). *Nucleic Acids Res* 50, W216-W221 (2022).
