# Supplementary material for: Combined and differential roles of ADD domains of DNMT3A and DNMT3L on DNA methylation landscapes in mouse germ cells
Source: Nat Commun. 2024 Apr 16;15:3266. doi: 10.1038/s41467-024-47699-2 (PMC11021467; doi:10.1038/s41467-024-47699-2)
Supplement: Supplementary file 3 — Description of Additional Supplementary Files [file 41467_2024_47699_MOESM3_ESM.pdf]

Combined and differential roles of ADD domains of DNMT3A and DNMT3L on DNA methylation landscapes in mouse germ cells

Naoki Kubo[1]*, Ryuji Uehara[1], Shuhei Uemura[1,3], Hiroaki Ohishi[2], Kenjiro Shirane[3] and Hiroyuki Sasaki[1]*

Description of Supplementary files

Supplementary Data 1. Oligonucleotides used to generate mutant mouse.

Supplementary Data 2. Summary of WGBS.

Supplementary Data 3. Summary of RNA-seq.

Supplementary Data 4. List of differentially expressed genes in the mutated FGOs.

Supplementary Data 5. List of high non-CG methylation regions in [*Dnmt3a*$^{ADD/ADD}$, *Dnmt3L*$^{ADD/ADD}$] FGOs and spermatozoa.

Supplementary Movie 1. Wild type spermatozoa.

Supplementary Movie 2. *Dnmt3a*$^{+/+}$ *Dnmt3L*$^{ADD/ADD}$ spermatozoa.

Supplementary Movie 3. *Dnmt3a*$^{ADD/ADD}$ *Dnmt3L*$^{+/+}$ spermatozoa.

Supplementary Movie 4. *Dnmt3a*$^{ADD/ADD}$ + *Dnmt3L*$^{ADD/ADD}$ spermatozoa.

(These movies are related to Supplementary Figure 1e)
