# Supplementary material for: Combined and differential roles of ADD domains of DNMT3A and DNMT3L on DNA methylation landscapes in mouse germ cells
Source: Nat Commun. 2024 Apr 16;15:3266. doi: 10.1038/s41467-024-47699-2 (PMC11021467; doi:10.1038/s41467-024-47699-2)
Supplement: Supplementary file 13 — Reporting Summary [file 41467_2024_47699_MOESM13_ESM.pdf]

nature portfolio

Corresponding author(s): Naoki Kubo, Hiroyuki Sasaki

Last updated by author(s): Mar 31, 2024

Reporting Summary

Nature Portfolio wishes to improve the reproducibility of the work that we publish. This form provides structure for consistency and transparency in reporting. For further information on Nature Portfolio policies, see our Editorial Policies and the Editorial Policy Checklist.

Statistics

For all statistical analyses, confirm that the following items are present in the figure legend, table legend, main text, or Methods section.

n/a	Confirmed	
☐	☒	The exact sample size (*n*) for each experimental group/condition, given as a discrete number and unit of measurement
☐	☒	A statement on whether measurements were taken from distinct samples or whether the same sample was measured repeatedly
☐	☒	The statistical test(s) used AND whether they are one- or two-sided<br>*Only common tests should be described solely by name; describe more complex techniques in the Methods section.*
☒	☐	A description of all covariates tested
☐	☒	A description of any assumptions or corrections, such as tests of normality and adjustment for multiple comparisons
☐	☒	A full description of the statistical parameters including central tendency (e.g. means) or other basic estimates (e.g. regression coefficient) AND variation (e.g. standard deviation) or associated estimates of uncertainty (e.g. confidence intervals)
☐	☒	For null hypothesis testing, the test statistic (e.g. *F*, *t*, *r*) with confidence intervals, effect sizes, degrees of freedom and *P* value noted<br>*Give P values as exact values whenever suitable.*
☒	☐	For Bayesian analysis, information on the choice of priors and Markov chain Monte Carlo settings
☒	☐	For hierarchical and complex designs, identification of the appropriate level for tests and full reporting of outcomes
☒	☐	Estimates of effect sizes (e.g. Cohen's *d*, Pearson's *r*), indicating how they were calculated

Our web collection on statistics for biologists contains articles on many of the points above.

Software and code

Policy information about availability of computer code

Data collection	No software was used for data collection
Data analysis	The software and versions used in this study are:<br>Bismark v0.20.0<br>bowtie v1.3.1<br>bedtools v2.25.0<br>STAR v2.5.3a<br>HTSeq 2.0.5<br>featureCounts v1.5.3<br>edgeR v3.14.0<br>RSAT Code version: Feb 16 17:12:44 2023<br>DAVID v2022q1

For manuscripts utilizing custom algorithms or software that are central to the research but not yet described in published literature, software must be made available to editors and reviewers. We strongly encourage code deposition in a community repository (e.g. GitHub). See the Nature Portfolio guidelines for submitting code & software for further information.

Data

Policy information about availability of data

All manuscripts must include a data availability statement. This statement should provide the following information, where applicable:

- Accession codes, unique identifiers, or web links for publicly available datasets
- A description of any restrictions on data availability
- For clinical datasets or third party data, please ensure that the statement adheres to our policy

All sequencing datasets generated in this study have been deposited in the Gene Expression Omnibus (GEO) under accession code GSE238228 [https://www.ncbi.nlm.nih.gov/geo/query/acc.cgi?&acc=GSE238228]. The WGBS data from Dnmt3aADD/ADD FGO is available in the Sequence Read Archive database (accession code PRJDB12492 [https://www.ncbi.nlm.nih.gov/bioproject/?term=PRJDB12492]). The WGBS data of Dnmt3a and Dnmt3L knockout FGOs and wild-type and Dnmt3L knockout spermatogonia at postnatal day 10 that were used in this study are available in DDBJ/GenBank/EMBL (accession code DRA000570 [https://ddbj.nig.ac.jp/resource/sra-submission/DRA000570]) and GEO (accession code GSE84140 [https://www.ncbi.nlm.nih.gov/geo/query/acc.cgi?acc=GSE84140]), respectively. H3K36me3 ChIP-seq data in FGOs and round spermatogonia that were also used in this study are available in GEO under accession codes GSE183969 [https://www.ncbi.nlm.nih.gov/geo/query/acc.cgi?acc=GSE183969] and GSE108717 [https://www.ncbi.nlm.nih.gov/geo/query/acc.cgi?acc=GSE108717], respectively. The mouse reference genome data (mm10) and the SNP information for the JF-1 mouse genome are available from https://hgdownload.cse.ucsc.edu/goldenpath/mm10/ and https://molossinus.brc.riken.jp/pub/For_Seq_Analysis/list_of_variations/, respectively.

Research involving human participants, their data, or biological material

Policy information about studies with human participants or human data. See also policy information about sex, gender (identity/presentation), and sexual orientation and race, ethnicity and racism.

Reporting on sex and gender	This study did not involve human participants, data, and material.
Reporting on race, ethnicity, or other socially relevant groupings	This study did not involve human participants, data, and material.
Population characteristics	This study did not involve human participants, data, and material.
Recruitment	This study did not involve human participants, data, and material.
Ethics oversight	This study did not involve human participants, data, and material.

Note that full information on the approval of the study protocol must also be provided in the manuscript.

Field-specific reporting

Please select the one below that is the best fit for your research. If you are not sure, read the appropriate sections before making your selection.

☒ Life sciences ☐ Behavioural & social sciences ☐ Ecological, evolutionary & environmental sciences

For a reference copy of the document with all sections, see nature.com/documents/nr-reporting-summary-flat.pdf

Life sciences study design

All studies must disclose on these points even when the disclosure is negative.

Sample size	No statistical method was used to pre-determine sample size. Practical considerations limited the number of samples that we could analyze.
Data exclusions	No data was excluded from the analysis.
Replication	For WGBS experiments, 2 biological replicates were performed. While wild-type spermatozoa dataset is produced from one replicate, the data was consistent with our previous published dataset. For RNA- seq data, 8 to 10 biological replicates were generated for each sample. All attempts of replications were successful.
Randomization	The experiments were not randomized. This study does not involve randomized samples.
Blinding	The investigators were not blinded to the group as no human subjects were involved and no subjective measurements were taken.

Reporting for specific materials, systems and methods

We require information from authors about some types of materials, experimental systems and methods used in many studies. Here, indicate whether each material, system or method listed is relevant to your study. If you are not sure if a list item applies to your research, read the appropriate section before selecting a response.

Materials & experimental systems

n/a	Involved in the study
☐	☒ Antibodies
☒	☐ Eukaryotic cell lines
☒	☐ Palaeontology and archaeology
☐	☒ Animals and other organisms
☒	☐ Clinical data
☒	☐ Dual use research of concern
☒	☐ Plants

Methods

n/a	Involved in the study
☒	☐ ChIP-seq
☒	☐ Flow cytometry
☒	☐ MRI-based neuroimaging

Antibodies

Antibodies used	antibodies against DNMT3A (NOVUS, 64B1446) for immunofluorescence staining (1:500 dilution) and western blotting (1:1000 dilution).<br>antibodies against DNMT3L (Abcam, ab194094) for immunofluorescence staining (1:500 dilution) and western blotting (1:1000 dilution).<br>antibodies against β-actin (Santa Cruz sc-69879) for western blotting (1:1000 dilution).<br>anti-rabbit IgG (H+L) CF 488A and anti-mouse IgG (H+L) CF 594 secondary antibodies (Biotium) for immunofluorescence staining (1:1000 dilution).<br>Horseradish peroxidase (HRP)-conjugated anti-mouse IgG and anti-rabbit IgG antibodies (Abcam, ab6789 and ab6721) for western blotting (1:30000 dilution).
Validation	DNMT3A antibody product information: https://www.novusbio.com/products/dnmt3a-antibody-64b1446_nb120-13888<br>DNMT3L antibody product information: https://www.abcam.com/en-is/products/primary-antibodies/anti-dnmt3l-antibody-epr18774-ab194094<br>β-actin antibody product information: chrome-extension://efaidnbmnnnibpcajpcglclefindmkaj/https://datasheets.scbt.com/sc-69879.pdf<br>anti-rabbit IgG (H+L) CF 488A product information: https://biotium.com/product/goat-anti-rabbit-igg-hl-highly-cross-absorbed/<br>anti-mouse IgG (H+L) CF 594 product information: https://biotium.com/product/donkey-anti-mouse-igg-hl-highly-cross-adsorbed/<br>HRP-conjugated anti-mouse IgG antibody product information: https://www.abcam.com/en-is/products/secondary-antibodies/goat-anti-mouse-igg-h-l-hrp-ab6789<br>HRP-conjugated anti-rabbit IgG antibody product information: https://www.abcam.com/products/secondary-antibodies/goat-rabbit-igg-hl-hrp-ab6721.html

Animals and other research organisms

Policy information about studies involving animals; ARRIVE guidelines recommended for reporting animal research, and Sex and Gender in Research

Laboratory animals	Mus musculus strains C57BL/6J and JF1 were used in this study. Embryos and 8 to 12 weeks old mice were used. Mice were group-housed in a specific-pathogen-free facility under standard housing conditions (12-h light/dark cycle, temperature 20-22°C, humidity 40-60%, and free access to water and food).
Wild animals	This study did not use wild animals.
Reporting on sex	This study described the findings of both male and female mice.
Field-collected samples	This study did not involve samples collected from the field.
Ethics oversight	All animal experiments were performed according to the ethical guidelines of Kyushu University and the protocols were approved by the Institutional Animal Care and Use Committee of Kyushu University (A22-087-1).

Note that full information on the approval of the study protocol must also be provided in the manuscript.

Plants

Seed stocks	This study did not use plants.
Novel plant genotypes	This study did not use plants.
Authentication	This study did not use plants.
